# Bottom-up vs. top-down connectivity imbalance in individuals with high-autistic traits: An electroencephalographic study

**DOI:** 10.3389/fnsys.2022.932128

**Published:** 2022-08-12

**Authors:** Mauro Ursino, Michele Serra, Luca Tarasi, Giulia Ricci, Elisa Magosso, Vincenzo Romei

**Affiliations:** ^1^Department of Electrical, Electronic, and Information Engineering “Guglielmo Marconi”, University of Bologna, Cesena, Italy; ^2^Centro Studi e Ricerche in Neuroscienze Cognitive, Dipartimento di Psicologia, Alma Mater Studiorum—Università di Bologna, Cesena, Italy; ^3^Istituto di Ricovero e Cura a Carattere Scientifico (IRCCS) Fondazione Santa Lucia, Rome, Italy

**Keywords:** autism spectrum disorder, Autistic Quotient, Granger causality, *in degree* and *out degree*, hubness and authority, bottom-up and top-down connections

## Abstract

Brain connectivity is often altered in autism spectrum disorder (ASD). However, there is little consensus on the nature of these alterations, with studies pointing to either increased or decreased connectivity strength across the broad autism spectrum. An important confound in the interpretation of these contradictory results is the lack of information about the directionality of the tested connections. Here, we aimed at disambiguating these confounds by measuring differences in directed connectivity using EEG resting-state recordings in individuals with low and high autistic traits. Brain connectivity was estimated using temporal Granger Causality applied to cortical signals reconstructed from EEG. Between-group differences were summarized using centrality indices taken from graph theory (*in degree*, *out degree*, *authority*, and *hubness*). Results demonstrate that individuals with higher autistic traits exhibited a significant increase in *authority* and *in degree* in frontal regions involved in high-level mechanisms (emotional regulation, decision-making, and social cognition), suggesting that anterior areas mostly receive information from more posterior areas. Moreover, the same individuals exhibited a significant increase in the *hubness* and *out degree* over occipital regions (especially the left and right pericalcarine regions, where the primary visual cortex is located), suggesting that these areas mostly send information to more anterior regions. *Hubness* and *authority* appeared to be more sensitive indices than the *in degree* and *out degree*. The observed brain connectivity differences suggest that, in individual with higher autistic traits, bottom-up signaling overcomes top-down channeled flow. This imbalance may contribute to some behavioral alterations observed in ASD.

## Introduction

Autism is a complex neurodevelopmental condition characterized by several behavioral peculiarities, involving avoidance of social interactions, reduced communication, and restricted interests [see the American Psychiatric Association [APA] (2022)]. The biological origin of this condition is a subject of active research, in an effort to understand its fundamental neural mechanisms. In this regard, a current perspective is that autistic traits could be explained by modifications in brain network characteristics, especially in the connectivity among brain areas underlying perception, social cognition, language, and executive functions (Kana et al., 2014).

Indeed, many recent studies have reported that individuals within the autism spectrum disorder (ASD) exhibit altered brain connectivity compared to typically developing individuals. However, literature reports are often inconsistent [see review papers by Maximo et al. (2014); Mohammad-Rezazadeh et al. (2016), Carroll et al. (2021)]. The traditional point of view, predominantly supported by studies using structural and functional MRI, hypothesizes that autism is characterized by long-range underconnectivity, potentially combined with local overconnectivity (Just et al., 2012; Abrams et al., 2013; Delbruck et al., 2019). Conversely, there have been several studies, using EEG and MEG, in which the hypoconnectivity hypothesis could not be confirmed in ASD. Rather, several studies pointed to hyperconnectivity among specific brain areas, especially between thalamic and sensory regions (Nair et al., 2013) or between the extrastriatal cortex, frontal and temporal regions (Murphy et al., 2012; Uddin et al., 2013; Fu et al., 2019). Finally, a third line of evidence points towards the existence of a more subtle mixture of hypo- and hyper-connectivity, suggesting the presence of multiple mechanisms (Di Martino et al., 2011; Lynch et al., 2013; Kana et al., 2014; Abbott et al., 2018).

Some of these differences, of course, can derive from methodological issues. Connectivity is an elusive concept that can be dramatically affected by the measurement technique adopted (for instance, fMRI vs. EEG/MEG), by the particular task involved (vs. resting state analysis), and perhaps more importantly, by the specific measure employed to estimate the connection strength (e.g., functional, effective or anatomical connectivity, directed or undirected measures, bivariate or multivariate). Indeed, most connectivity measures in literature are not-directional and hence are inadequate to discover differences in lateralization or in top-down vs. bottom-up information processing (O’Reilly et al., 2017).

In particular, it is well-known that cognitive functions are characterized by a complex balance between integration, involving the coordination among several brain areas, and segregation, involving specialized computations in local areas. According to the predictive coding theory (Clark, 2013), the brain continually generates models of the world by integrating data coming from sensory input with information from memory. Sensory perception is thus the result of a combination between present data from the external world (usually carried by feedforward bottom-up connectivity) and past or prior knowledge (mainly conveyed through feedback, top-down connections); hence, an equilibrium between these directional connectivity patterns is necessary to adaptatively integrate stimuli-driven and internally-driven representations, preventing their segregation or excessive bias towards one or the other.

Recent hypotheses (Pellicano and Burr, 2012; Van de Cruys et al., 2014) assume that ASD individuals exhibit an impaired predictive coding, characterized by an imbalance between these two processing streams, i.e., dominant bottom-up processing and relatively weaker top-down influences compared with control individuals. This signifies that people in the autistic spectrum would pose much more emphasis on present sensory stimuli and somewhat less weight on contextual information. This imbalance, in turn, may result in poor social adaptation and insufficient appropriateness to social requirements (Sinha et al., 2014). Results that support this point of view include a reduced susceptibility to illusions and top-down expectations (Skewes et al., 2015; Crespi and Dinsdale, 2019) and increased local (vs. global) processing in individuals within the autism spectrum (Mottron et al., 2006; Cribb et al., 2016) leading to a more stimulus- and detail-driven perceptual style.

The aforementioned alterations in predictive coding may be caused by altered brain connectivity, especially concerning top-down vs. bottom-up circuitry (Tarasi et al., 2022). Additionally, alterations in connectivity patterns may involve a different transmission of brain rhythms and an impaired wave synchronization, which plays a pivotal role in several cognitive tasks, including attention, information selection, working memory, and emotion (Basar-Eroglu et al., 2007; Clayton et al., 2015).

Finally, increasing evidence both at the genetic and behavioral levels demonstrates that autism does not represent a dichotomy condition (i.e., one ON/OFF in type) but is best described as a spectrum of manifestations ranging from clinical forms to trait-like expressions within the general population (Baron-Cohen et al., 2001; Cribb et al., 2016; Bralten et al., 2018) that share a peculiar cognitive style that distinguishes them from the rest of the clinical and nonclinical population (Tarasi et al., 2022).

Following these ideas, in a recent paper (Tarasi et al., 2021), we investigated whether the patterns of brain connectivity, estimated with Granger causality from EEG source reconstruction, exhibit differences in two nonclinical groups classified as low or high on autistic traits. Preliminary results suggested that connectivity along the fronto-posterior axis is sensitive to the magnitude of the autistic features and that a prevalence of ascending connections characterized participants with higher autistic traits.

The present study aims to further extend the previous work on a larger cohort allowing for an improved connectivity analysis by implementing measures taken from the graph theory. In particular, new aspects of the present study concern: (i) the use of a larger data set; (ii) a preliminary analysis at the lobe level; (iii) the use of more sophisticated indices taken from the graph theory, such as hubness and authority; (iv) the use of a more sophisticate statistical analysis (i.e., the use of sparse connectivity matrices) to better point out differences in connectivity between the two groups.

Particularly, graph theory represents a powerful tool able to summarize complex networks consisting of hundreds of edges, using a few parameters with a clear geometrical meaning. Recently, this theory has been applied with increasing success as an integrative approach, able to evaluate the complex networks that mediate brain cognitive processes (van Wijk et al., 2010; Wang, 2010; Minati et al., 2013; Farahani et al., 2019). In particular, since our attention here is primarily devoted to the presence of differences in the direction of connections (ascending vs. descending, lateralization, etc.), we focused our analysis on the *in degree* and *out degree*, defined as the sum of connection strengths entering or leaving a given node. Furthermore, we also tested whether two analogous but more specialized measures of centrality, *hubness* and *authority*, can provide additional information to better characterize directionality. The hub’s index of a node is the weighted sum of the authority’s indices of all its successors; hence, this measure summarizes the capacity of a node to send information to other critical, authoritative nodes. The authority’s index of a node is the weighted sum of the hub’s indices of all its predecessors and summarizes the capacity of a node to receive essential information from hubs. Here, we investigate whether differences in these measures, and the pattern of *out* and *in* connections from the dominant nodes, can reveal a difference in the network’s topology, and alterations in information processing, as a function of the autistic trait.

## Materials and methods

### Participants

Forty participants (23 female; age range 21–30, mean age = 24.1, SD = 2.4), with no neurocognitive or psychiatric disorders, took part in the study. All participants signed a written informed consent before taking part in the study, conducted according to the Declaration of Helsinki and approved by the Bioethics Committee of the University of Bologna. All participants completed the Autism-Spectrum Quotient test (AQ) (Baron-Cohen et al., 2001). The mean AQ score was 16.1 ± 6.6. The AQ is a self-report widely used to measure autistic traits in the general population. It provides a global score, with higher values indicating higher levels of autistic traits. We used the original scoring methods converting each item into a dichotomous response (agree/disagree) and assigning the response a binary code (0/1). In the present study, the total score of the AQ was considered, and the Italian version of the AQ was adopted (Ruta et al., 2012). The participants were divided into two groups, depending on their AQ score being below or above a given cutoff, with the cutoff set to 17, since this value corresponds to the average AQ score in the non-clinical population (Ruzich et al., 2015). In the following, we will refer to the two groups of participants as Low AQ score Group (*N* = 21) and High AQ score Group (*N* = 19).

### EEG acquisition and preprocessing

Participants comfortably sat in a room with dimmed lights. Electroencephalographic activity (EEG) was recorded at rest for 2 min while participants kept their eyes closed. A set of 64 electrodes was mounted according to the international 10–10 system. EEG was measured with respect to a vertex reference (Cz), and all impedances were kept below 10 kΩ. EEG signals were acquired at a rate of 1000 Hz. EEG was processed offline with custom MATLAB scripts (version R2020b) and the EEGLAB toolbox (Delorme and Makeig, 2004). The EEG recording was filtered offline in the 0.5–70 Hz band. The signals were visually inspected, and noisy channels were spherically interpolated. An average of 0.05 ± 0.15 channels were interpolated. The recording was then re-referenced to the average of all electrodes. Subsequently, we applied the Independent Component Analysis (ICA), an effective method largely employed to remove EEG artifacts. In particular, we removed the EEG recording segments corrupted by noise through visual inspection and then we removed all the independent components containing artifacts clearly distinguishable by means of visual inspection from brain-related components. An average of 3 ± 3.7 independent components were removed for each participant.

### Cortical sources reconstruction and regions of interest definition

Since we were interested in connectivity analysis, cortical source activity was reconstructed from pre-processed EEG signals. To this aim, intracortical current densities were estimated using the Matlab toolbox Brainstorm (Tadel et al., 2011). Firstly, to solve the forward problem, a template head model based on realistic anatomical information (ICBM 152 MNI template) was used. The model consists of three layers representing the scalp, the outer skull surface, and the inner skull surface, and includes the cortical source space discretized into 15,002 vertices. The forward problem was solved in OpenMEEG software (Gramfort et al., 2010) *via* the Boundary Element Method.

sLORETA (standardized Low-Resolution Electromagnetic Tomography) algorithm was used for cortical sources estimation. sLORETA is a functional imaging technique belonging to the family of linear inverse solutions for 3D EEG distributed source modeling (Pascual-Marqui, 2002). Specifically, this method computes a weighted minimum norm solution, where localization inference is based on standardized values of the current density estimates. The solution provided is instantaneous, distributed, discrete, linear with the property of zero dipole-localization error under ideal (noise-free) conditions. Constrained dipole orientations were chosen for sources estimation, modeling each dipole as oriented perpendicularly to the cortical surface. Hence, for each participant, we reconstructed the resting-state time series of standardized current densities at all 15,002 cortical vertices.

Then, the cortical vertices were grouped into cortical regions according to the Desikan–Killiany atlas (Desikan et al., 2006) provided in Brainstorm, which defines 68 regions of interest (ROIs). The activities of all vertices belonging to a particular ROI were averaged at each time point, obtaining a single time series representative of the activity of that cortical ROI. It is worth noticing that, by considering the average behavior at the ROIs level, it was possible to mitigate some possible inaccuracies in source reconstruction at single vertex level, due to the use of a template head model for all participants (instead of subject-specific head models).

Table 1 lists the 68 Desikan-Killiany ROIs and provides the mapping of individual ROIs to each lobe.

**TABLE 1:** The approximate mapping of the “Desikan-Killiany” ROIs to the lobes.

ROI	Label	Lobe	ROI	Label	Lobe
Banks of Sup. Temp. Sulcus	BK	Temporal	Parahippocampal	PH	Temporal
Caudal anterior cingulate	cAC	Frontal	Pars opercularis	pOP	Frontal
Caudal middle frontal	cMF	Frontal	Pars orbitalis	pOR	Frontal
Cuneus	CU	Occipital	Pars triangularis	pTR	Frontal
Entorhinal	EN	Temporal	Pericalcarine	PCL	Occipital
Frontal pole	FP	Frontal	Postcentral	POC	Parietal
Fusiform	FU	Temporal	Posterior cingulate	PCG	Parietal
Inferior parietal	IP	Parietal	Precentral	PRC	Frontal
Inferior temporal	IT	Temporal	Precuneus	PCU	Parietal
Insula	IN	Parietal	Rostral anterior cingulate	rAC	Frontal
Isthmus cingulate	IST	Parietal	Rostral middle frontal	rMF	Frontal
Lateral occipital	LO	Occipital	Superior frontal	SF	Frontal
Lateral orbitofrontal	lOF	Frontal	Superior parietal	SP	Parietal
Lingual	LG	Occipital	Superior temporal	ST	Temporal
Medial orbitofrontal	mOF	Frontal	Supramarginal	SMG	Parietal
Middle temporal	MT	Temporal	Temporal pole	TP	Temporal
Paracentral	PAC	Frontal	Transverse temporal	TT	Temporal

The Desikan–Killiany atlas comprises 34 ROIs in each hemisphere. The mapping proposed by FreeSurfer (https://surfer.nmr.mgh.harvard.edu/fswiki/CorticalParcellation) was used as a reference. The only difference between our mapping and the reference resides in the mapping of the insula, which was not ascribed to any lobe in FreeSurfer. We assigned the insula to the parietal lobe.

### Granger causality analysis

Once the time waveform in each cortical ROI was estimated (as described above), for each participant *k* (*k* = 1,…,40) we evaluated the connectivity among the ROIs. To this aim, we adopted Granger Causality (GC) (Granger, 1969; Geweke, 1982; Ding et al., 2006; Bressler and Seth, 2011; Stokes and Purdon, 2017) which provides directional metrics of connectivity, and is based on the autoregressive (AR) modeling framework as described in the following.

Let’s indicate with *x*_*k*,*i*_[*n*] and *x*_*k*,*j*_[*n*] two temporal series representing the activity of two distinct cortical ROIs (*ROI*_*i*_ and *ROI*_*j*_) for participant *k*, where *n* is the discrete time index. The Granger Causality quantifies the causal interaction from *ROI*_*i*_ to *ROI*_*j*_ as the improvement in predictability of *x*_*k*,*j*_[*n*] at time sample *n* when using a bivariate AR representation, including both past values of *x*_*k,j*_ and past values of *x*_*k,i*_, compared to a univariate AR representation, including only past values of *x*_*k,j*_. Mathematically, the following two equations hold for the univariate and bivariate AR model, respectively.


(1) $$x_{k,j}\,\left[n\right]=\sum_{m=1}^{p}a_{k,j}\,\left[m\right]\,x_{k,j}\,\left[n-m\right]+\eta_{k,j}\,\left[n\right]$$

(2) $$x_{k,j}\,\left[n\right]=\sum_{m=1}^{p}b_{k,j}\,\left[m\right]\,x_{k,j}\,\left[n-m\right]$$

$$\;\;+\sum_{m=1}^{p}c_{k,j\,i}\,\left[m\right]\,x_{k,i}\,\left[n-m\right]+\varepsilon_{k,j}\,\left[n\right]$$

Index *m* represents the time lag (in time samples), and *p* (model order) defines the maximum time lag, i.e., the maximum number of lagged observations included in the models. Thus, in Eq. 1, the current value of *x*_*k,j*_ (at time sample *n*) is predicted in terms of its own *p* past values (at time samples *n*−1,*n*−2,…,*n*−*p*), while in Eq. 2 prediction is made also in terms of the *p* past values of *x*_*k,i*_. *a*, *b*, *c* are the model’s coefficients (dependent on time lag), and the time series η_*k*,*j*_[*n*] and ε_*k*,*j*_[*n*] represent the prediction error of the univariate and bivariate AR model, respectively. The prediction error variance quantifies the model’s prediction capability based on past samples: the lower the variance, the better the model’s prediction. The GC from *x*_*k,i*_ to *x*_*k,j*_ is defined as the logarithm of the ratio between the variances of the two prediction errors, i.e.,


(3) $$G\,C_{k,R\,O\,I_{i}\to R\,O\,I_{j}}=\ln\frac{v\,a\,r\,\left\{\eta_{k,j}\,\left[n\right]\right\}}{v\,a\,r\,\left\{\varepsilon_{k,j}\,\left[n\right]\right\}}$$

The measure in Eq. 3 is always positive: the larger its value, the larger the improvement in *x*_*k*,*j*_[*n*]prediction when using information from the past of *x*_*k,i*_ together with the past of *x*_*k,j*_, and this is interpreted as a stronger causal influence from *ROI*_*i*_ to *ROI*_*j*_. Similarly, Granger Causality from *x*_*k,j*_ to *x*_*k,i*_, *GC*_*k*,*ROI*_*j*_→*ROI*_*i*__, is computed *via* the same procedure, building the AR models for the time series *x*_*k,i*_.

For each participant *k*, we computed the two directed measures of GC for each pair of ROIs, overall obtaining 68×68 connectivity values (with all auto-loops equal to zero). In all cases, the order *p* of the AR models was set equal to 20, corresponding to 20 ms time span at 1000 Hz sampling rate (as in our data); thus, in this study, the functional interactions between nodes were evaluated within 20 ms time delay. This value for parameter *p* was determined based on a preliminary analysis where we tested different values for the order of the model, obtaining that GC results did not change substantially for *p*≥20.

### Indices derived from graph theory

As previously reported by other authors (Deshpande et al., 2009; Sporns, 2018) the connectivity between the ROIs of a brain network can be described as a weighted graph, where the magnitude of the connectivity between two ROIs is represented as the weight of an edge, whilst the ROIs connected by the edge are the nodes of the graph. A most remarkable consequence of the adoption of this representation for the brain network is the introduction of several concepts and measures from Graph Theory, which allows us to achieve a better understanding of the network’s topology (van Wijk et al., 2010; Minati et al., 2013; Farahani et al., 2019). For this study, we focused on centrality indices that take into account the direction of connections, specifically *authority*, *hubness*, *in degree*, and *out degree* centralities. These indices, which will be detailed in the following, were specifically selected for their focus on the ROIs’ inputs and outputs, which we hypothesized could offer confirmatory evidence of connectivity patterns previously observed in individuals with low and high autistic traits (Tarasi et al., 2021).

#### The graph

A graph is the mathematical abstraction of the relationships between some entities. The entities connected in a relationship are called “nodes” of the graph and are often represented graphically in the form of points. These nodes are connected by edges. While the simplest form of a graph is undirected (i.e., the edges do not have orientation), the graph we use to describe a brain network is a weighted directed graph (or digraph), i.e., it has oriented edges, each one with a weight representing the strength of the connection.

To obtain the graphs, for each participant the connectivity matrix was normalized so that its elements provided a sum of 100 (i.e., each connectivity value was divided by the total sum of connections and multiplied by 100). Furthermore, the normalized 68×68 matrices (which we will be calling “complete” matrices for clarity) were turned into 68×68 sparse matrices by removing (i.e., setting to zero) any connection that was not significantly different between the High and Low AQ score Groups. In particular, a two-tailed Monte-Carlo testing was applied (5,000 permutations) and, based on its results, not significant connections were defined as having an uncorrected p-value greater than 0.05.

Forty graphs (one per participant) were obtained both for the complete normalized and the sparse matrices. For each of these graphs, centrality indices were then computed. Although a preliminary investigation was performed on the complete matrices, our analysis is mainly focused on sparse matrices since by excluding “similar” connections we expect to better capture differences in the connectivity patterns and in graph indices between the two groups.

#### Centrality indices

Graph theory defines a multitude of indices and coefficients that allow describing the topology of a network from different points of view. Centrality indices are part of these. They measure the importance of a particular node in the network. The four centrality indices considered in this study (*in degree*, *out degree*, *authority*, *hubness*) quantify the importance of a node as a source or a sink for the edges. In the following, we will first introduce the *in degree* and *out degree* centralities; then, *authority* and *hubness* will be described, stressing on how they differ from *in degree* and *out degree*.

In the following, *A* will always indicate a generic adjacency matrix (i.e., a matrix containing all edges’ weights). In particular, the element *A*_*i,j*_ of the matrix will represent the weight of the edge connecting node *i* to node *j*.

*In degree* is the sum of the weights of the edges entering into a node.


(4) $$I\,n\,\;\,d\,e\,g\,r\,e\,e_{i}=\sum_{j}A_{j,i}$$

*Out degree* is the sum of the weights of the edges exiting from a node.


(5) $$O\,u\,t\,\;\,d\,e\,g\,r\,e\,e_{i}=\sum_{j}A_{i,j}$$

As a result of their direct dependence on the strength of input and output connections, *in degree* and *out degree* provide an immediate description of the nodes most involved in the transmission (*out degree*) and reception (*in degree*) of information.

*Authority* and *hubness* centralities include a more refined concept compared with *in degree* and *out degree* centralities and have a distinctive feature of strict interdependence. Their mathematical formulation is the following one.

*Authority* (*x_i_*) is proportional to the sum of the weights of edges entering a node, multiplied by the *hubness* of the node the edge originates from.


(6) $$x_{i}=\alpha \,\sum_{j}A_{j,i}\,y_{j}$$

*Hubness* (*y_i_*) is proportional to the sum of the weights of edges exiting from a node, multiplied by the *authority* of the node the edge points to.


(7) $$y_{i}=\beta \,\sum_{j}A_{i,j}\,x_{j}$$

These indices were computed using the function provided by the Matlab’s libraries contained in the Category “Graph and network algorithms” (Matlab R2021a), particularly the command “digraph/centrality.” This function sets both α and β equal to 1 and calculates *authority* and *hubness via* an iterative procedure.

Similar to *in degree* and *out degree*, *hubness* and *authority* provide a measure about which nodes of the network are primarily involved in the transmission (*hubness*) and reception (*authority*) of information, but they also mutually account for the centrality of the receiving and sending nodes. In particular, since these two centrality indices point to each other (i.e., to compute *authority*, we use *hubness*, and vice versa), they imply that strong connections exist between nodes with high *authority* and nodes with high *hubness*, and these indices may be useful to further emphasize any existing directionality in the connectivity pattern.

#### Connectivity analysis

For each participant, starting from either the complete normalized or the sparse 68×68 matrix, the four centrality indices were computed at each of the 68 ROIs. Additionally, we computed the average complete and sparse connectivity matrix in the Low AQ score Group and in the High AQ score Group, and then their difference.

Initially, we performed an analysis at the level of macro regions (englobing several ROIs) rather than at single ROI level. To this aim, we considered 8 regions corresponding to brain lobes (frontal, parietal, temporal, and occipital lobes, both left and right). Specifically, for each participant, the 68×68 connectivity matrix was transformed into an 8×8 connectivity matrix; the elements of the 8×8 matrix were filled in with the sum of all the connections going from one lobe to another. The elements of the 8×8 matrices were subsequently tested for statistical significance across the two groups of participants, by applying a two-tailed *t*-test (significance level 0.05, no correction), resulting in 64 comparisons. Furthermore, the 8×8 difference matrix was computed, by subtracting the 8×8 mean connectivity matrix of the Low AQ score Group from the 8×8 mean connectivity matrix of the High AQ score Group. Thus, the elements of the difference matrix greater than 0 represented stronger connectivity for the High AQ score Group, while elements of the difference matrix less than 0 represented stronger connectivity for the Low AQ score Group.

Then, a more detailed analysis was performed at the level of each ROI.

A first analysis was performed on the complete normalized connectivity matrix to understand the Granger flow in some key regions. Normalization of the connectivity matrix was necessary to avoid the presence of a few individuals with higher connectivity strongly affects the final results.

In particular, we computed the authority and the hubness of each ROI in each individual subject, and evaluated the correlation between these centrality indices and the AQ score. In this way, we identified the ROIs which exhibit a significant correlation between the centrality indices (in particular authority and hubness) and the AQ score. The *p*-value is computed by transforming the correlation to create a t-statistic having N-2 degrees of freedom, where N is the number of data points.

In the case of the sparse matrix, for each centrality index, we identified the ROIs that exhibited a significant statistical difference between the two groups. ROI’s significance was defined as a Bonferroni-corrected *p*-value less or equal to 0.05 where the p-value was obtained *via* Monte-Carlo testing.

Then, both in case of the complete and sparse matrix, once the significant ROIs were identified for each index, the connectivity differences between the Low and High AQ Score Group were plotted for the significant ROIs only, separately for each index (in particular in case of the *authority* index and *hubness* index); this serves to evidence differences between the two groups in the pattern of connections entering into *authority* nodes and exiting from *hub* nodes.

## Results

### Analysis of the complete connectivity matrix

#### Lobes’ analysis

**FIGURE 1:**
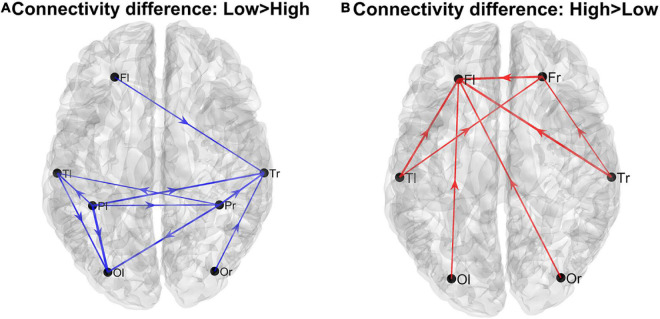
Patterns of the main connection differences linking the four lobes (Frontal left and right, Fl and Fr, Temporal left and right, Tl and Tr, Parietal left and right, Pl and Pr, Occipital left and right, Ol and Or). The left panel **(A)** describes connections differences which are higher in the Low AQ score Group, while the right panel **(B)** describes connections differences which are higher in the High AQ score Group. Only connections differences with |*t*| > 1 (student *t*-test) are plotted.

Using the complete connectivity matrix, the connection difference between the two groups does not reach a significativity level. Hence the following results can only be considered just as a preliminary exploratory analysis, and connection differences can be only regarded as indicative of a main flow pattern in the two groups. The results are illustrated in Figure 1, where we show only the connection differences with |*t*| > 1 (which corresponds to a *p* < 0.15 in the case of a one-tailed student *t*-test). Higher blue lines denote connectivity higher in the Low AQ score Group (left panel), and red lines connectivity higher in the High AQ score Group (right panel). Results show that left to right connections (i.e., entering into the right temporal lobe) were higher in the Low AQ score Group; conversely, connectivity was mainly bottom-up (i.e., entering into the frontal lobes) in the High AQ Score Group.

#### Analysis on the individual regions of interests

**FIGURE 2:**
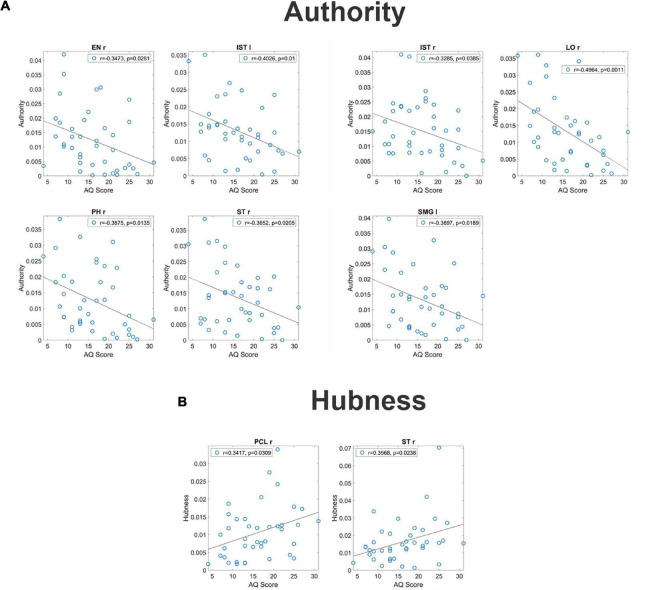
Correlation between the authority and the AQ score [upper panel **(A)**] and correlation between the hubness and the autistic score [bottom panel **(B)**] for all ROIs which exhibit a significant *p*-value (uncorrected) for the correlation. These correlations have been computed on the complete normalized connectivity matrix. It is worth noting that the correlation is negative for the authority, denoting a more significant input flow in the Low AQ score Group, while correlation is positive for the hubness, denoting a more significant output flow for the High AQ score Group.

For what concerns authority, seven regions (EN r, IST l, IST r, LO r, PH r, ST r, and SMG l) exhibited a significant correlation between the AQ score and authority (see Figure 2, upper panels). It is worth-noting that, in all these ROIs, correlation was negative signifying that authority increased in subjects with smaller autistic traits.

For what concerns hubness, only two regions (PCL l and ST r) exhibited a significant correlation between the AQ score and hubness; in both cases, the correlation was positive, signifying that hubness increased with the autistic traits (see Figure 2 bottom panels).

**FIGURE 3:**
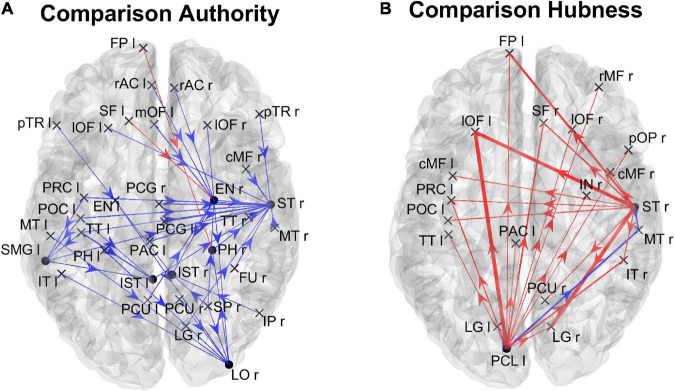
Patterns of the main connection difference which exit from the ROIs with a significant correlation between authority and the AQ score [left panel **(A)**] and which enters into the ROIs with a significant correlation between the hubness and the AQ score [right panel **(B)**]. Blue lines denote correlation differences that are higher in the Low AQ score group, and red lines connections which are higher in the High AQ score group. Only connection differences higher than 0.015 on the complete connectivity matrix have been plotted. Three levels of thickness are adopted, with a larger thickness indicating a larger connectivity difference.

The left panel in Figure 3 shows the main connections differences entering into the seven regions (EN r, IST l, IST r, LO r, PH r, ST r, and SMG l) whose authority was significantly correlated with the AQ score. The right panel shows the main connection differences exiting from the two regions (PCL l and ST r) whose hubness was significantly correlated with AQ score. Blue lines denote higher connectivity for the Low AQ score Group, red lines higher connectivity for the High AQ score Group. Since we are working with a complete connection matrix, only connection differences above a given threshold (threshold = 0.015) are plotted to simplify the figure. In particular, since all connectivity matrices are normalized to 100, and we have a total number of 68×67 connections, the average value of each connection is 0.021. The previous threshold approximately corresponds to the difference between one connection increased 33% above the mean value, and another connection reduced by 33% below the mean value (i.e., 66% of the mean).

The figure shows that the majority of connections entering the authority regions were stronger in the Low AQ score Group (as expected from the previous analysis), and these connections were mainly top-down in type (especially entering into the LO r) and left to right (especially entering into the EN r and the ST r). Conversely, the majority of connections exiting from the two hubs, PCL l and ST r, were stronger in the High AQ score Group (as expected from the previous analysis), with a bottom-up connectivity, especially emerging from the PCL l, and right-to left from ST r. These results are coherent with those at lobe level displayed in Figure 1.

### Analysis on the sparse connectivity matrix

The previous analysis, accomplished on the overall normalized connectivity matrix, pointed out the presence of some authority nodes especially involved in top-down and left-to-right connectivity for the low-autistic trait population, and some hubness nodes characterized by bottom-up and right-to-left connectivity for the high-autistic trait population. The difficulty in the use of a complete connectivity matrix, however, derives from the presence of many connections with no clear statistical difference between the two groups. This is reflected in the poor statistical significance of the connection difference and, for what concerns the correlation, in a *p*-value that, although significant, cannot survive the statistical correction. This means that the previous results can be considered as a mere hypothesis generated from data, requiring further more complete validation.

For this reason, in order to better unmask differences, in the following a different analysis is presented, by focusing attention only on the connections which exhibited a significant statistical difference in the two groups. Hence, as described in the Section “Materials and methods,” we consider sparse connectivity matrices. This kind of analysis has the benefit of revealing a greater number of regions with statistical differences in connection flow.

#### Lobes’ analysis

**FIGURE 4:**
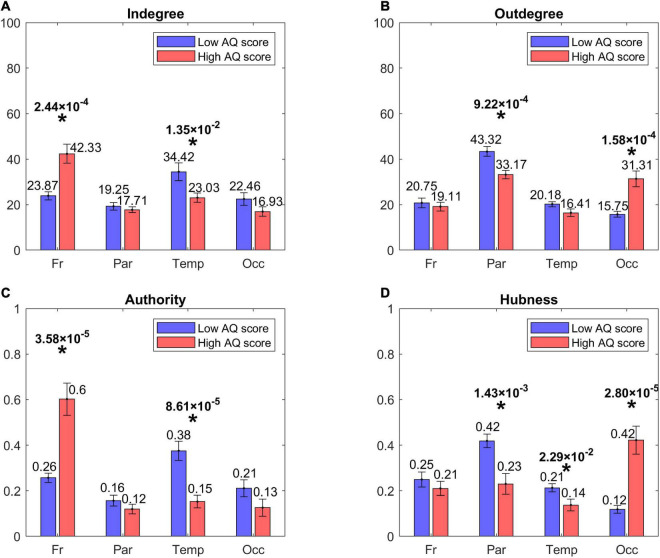
Bar plots representing the centrality indices [in degree: panel **(A)**, out degree: panel **(B)**, authority: panel **(C)**, hubness: panel **(D)**] for the four lobes of the brain, i.e., Frontal (Fr), Parietal (Par), Temporal (Temp), and Occipital (Occ) in each group of participants (red bars for the High AQ score Group, blue bars for the Low AQ score Group). Each bar shows the index value (mean ± SEM) for the specific area in the specific group of participants. As per definition, the sum of the *authority* values and the sum of the *hubness* values across all areas provide a total of 1, while the sum of the *in degree* values and the sum of *out degree* values across all areas is equal to 100. The asterisks indicate the presence of a statistically significant difference between the two groups (*p* < 0.05, uncorrected).

Figure 4 shows the centrality indices (*in degree*, *out degree*, *authority*, *hubness*) computed at the level of the four lobes (frontal, parietal, temporal, and occipital) from the sparse matrix. The asterisks denote statistically significant differences between the two groups. As it is evident from the left panels, High AQ score individuals exhibited a statistically significant increase in the connections entering into the frontal regions, and this difference was even more marked if *authority* was used as a centrality measure instead of the *in degree*. Conversely, Low AQ score individuals exhibited more significant connections entering into the temporal regions; even in this case, the significance increased if the *authority* measure was used. For what concerns the connections emerging from regions (right panels), High AQ score individuals exhibited more significant connections emerging from the occipital regions, whereas Low AQ score individuals showed a higher significance in the parietal regions. For both emerging connection outcomes, the significance was more evident if *hubness*, instead of the *out degree* measure, was used.

**FIGURE 5:**
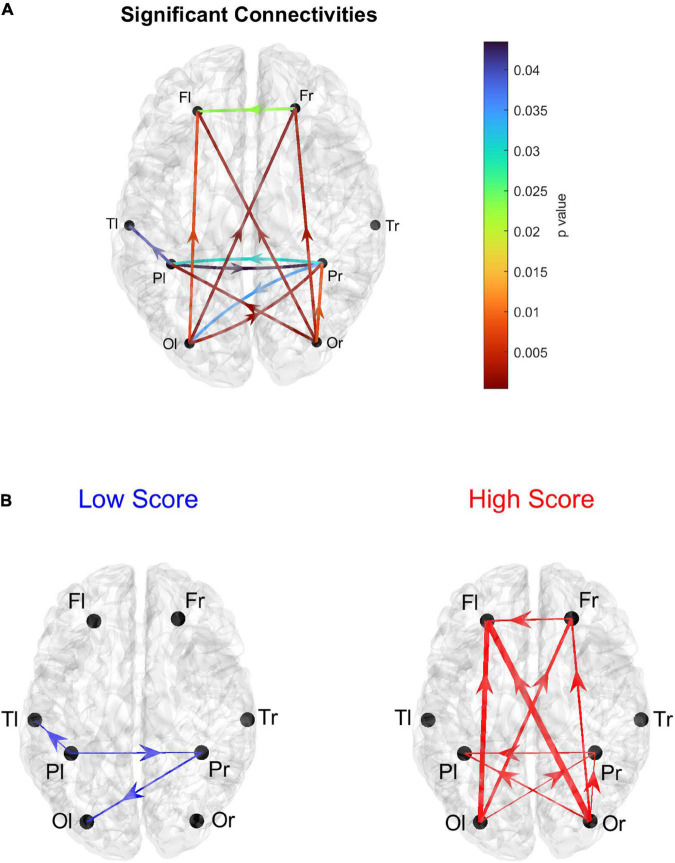
Representation of the connections linking the eight lobes of the brain, Frontal (F), Parietal (P), Temporal (T), and Occipital (O), considering separately the right (r) and left (l) hemispheres. Only the connections that exhibited a statistically significant difference between the two groups (*p* ≤ 0.05, uncorrected) are represented. The upper panel **(A)** shows the *p*-values of the significantly different connections. The lower panels **(B)** represent the differences in connectivity strength: the blue diagram (Low > High) shows the connection differences for those connections that resulted significantly stronger in the Low AQ score Group compared to the High AQ score Group; the red diagram (High > Low) shows the connection differences for those connections that resulted significantly stronger in the High AQ score Group compared to the Low AQ score Group. The thickness of each link varies according to the value of the connection difference. Three levels of thickness are adopted, with a larger thickness indicating a larger connectivity difference.

In order to further investigate the results arising from the above histograms, Figure 5 represents the statistically significant connections (i.e., those which exhibited significant differences between the two groups) linking the eight lobes of the brain; in this case, the homologous regions in the left and right hemisphere were considered separately. The upper panel displays the *p*-value of all significant connections using a color scale, while the bottom panel shows the connection differences (in red the connections which were significantly stronger in High AQ score individuals, in blue the connections significantly stronger in Low AQ score individuals). The results confirm those reported in Figure 4, showing that, in the High AQ score Group, significantly stronger connections were mainly directed from the occipital toward the frontal regions. The pattern in the Low AQ score Group showed significantly stronger connections emerging from the left parietal lobe, directed toward the right parietal, left temporal and left occipital regions.

#### Analysis of the individual regions of interest

**FIGURE 6:**
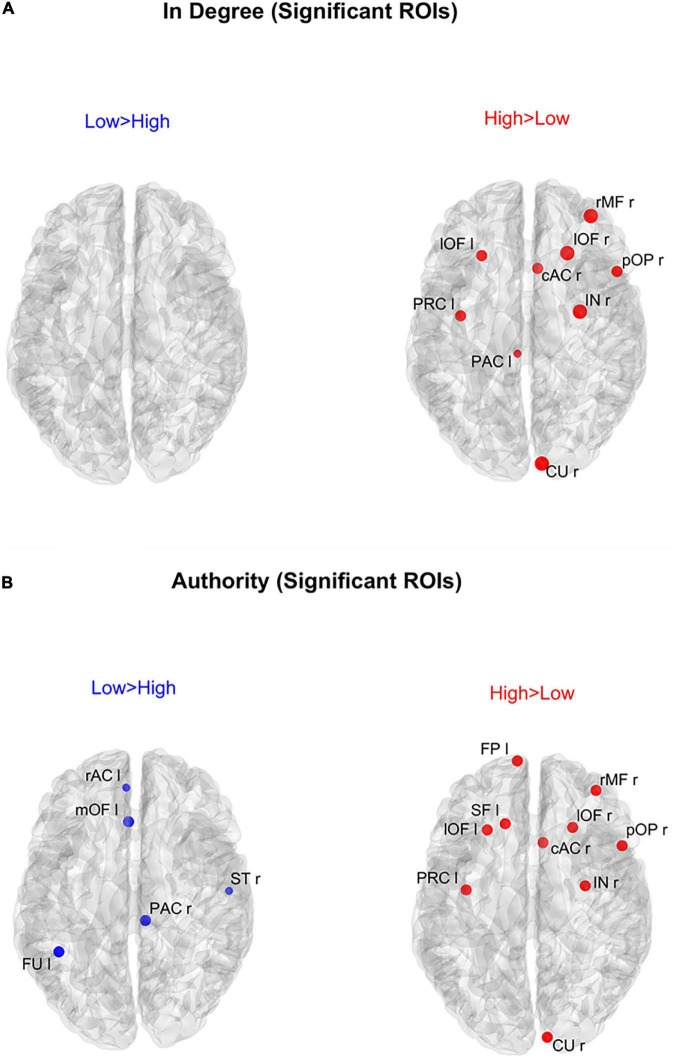
Positions of the ROIs which exhibited a significant difference in the *in degree* index [upper panels **(A)**] or in the *authority* index [lower panels **(B)**] between the two groups (*p*-value < 0.05, Bonferroni corrected). The left panels in blue (Low > High) display the ROIs having significantly higher centrality index in the Low AQ score Group compared to the High AQ score Group. The right panels in red (High > Low) display the ROIs having significantly higher centrality index in the High AQ score Group compared to the Low AQ score Group. The significant ROIs are shown as simple dots and represent regions to which important information enters. Three levels of dots’ size have been adopted: the larger the dot size, the more significant the centrality difference. For the panels where no dot appears over the brain map (i.e., *in degree* for Low > High), the constraint of significance was not satisfied by any of the 68 ROIs.

Figure 6 shows the positions of the ROIs which exhibited a significant difference (Bonferroni corrected) in the *in degree* (upper panels) and in the *authority* (bottom panels) indices between the two groups. The right upper panel evidences that in the High AQ score Group the *in degree* index was significantly higher (compared to the other group) especially in the frontal ROIs. This pattern was even more evident if *authority* index was used (bottom right panel). Conversely, the Low AQ score Group did not exhibit any appreciable increase in the *in degree* index, while some regions in the temporal, parietal and frontal lobes exhibited an increased *authority* without a clear topological organization.

**FIGURE 7:**
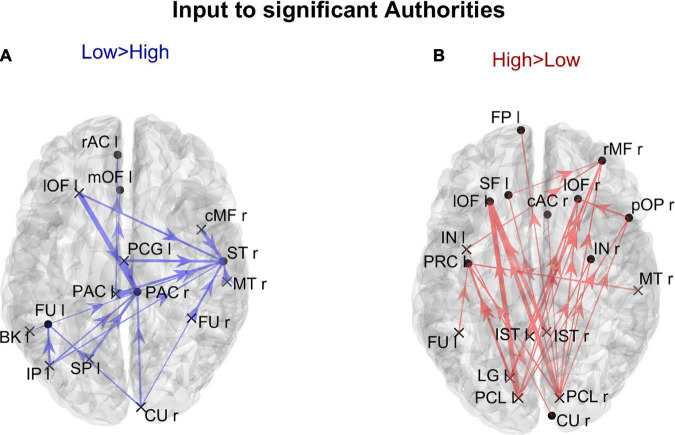
Representation of the connection differences *entering* into the ROIs which exhibited significant differences of *authority* between the two groups. The left panel in blue [Low > High, panel **(A)**] displays the connection differences entering into the “Low > High” *authority* ROIs (the ROIs shown in the left lower panel in Figure 6), for connections higher in the Low compared to the High AQ score Group. The right panel in red [High > Low, panel **(B)**] displays the connection differences entering into the “High > Low” *authority* ROIs (the ROIs shown in the right lower panel in Figure 6), for connections higher in the High compared to the Low AQ score Group. The plotted connections run from a generic output ROI (marked with a cross) toward the ROIs with significantly different authorities (marked with a dot). The thickness of each link varies according to the value of the connection difference. Three levels of thickness are adopted, with a higher thickness indicating a larger connectivity difference.

In order to gain a deeper understanding of the previous patterns (limited to *authority* only), Figure 7 shows the connection differences entering into all ROIs with significantly higher *authority* in either group. In the High AQ score Group, these connections mainly linked the two occipital regions PCL (right and left) toward frontal regions: particularly evident were the connections entering the two lOF (left and right), and the right rMF. Thus, a clear bottom-up pattern of connections emerged, supporting the results in Figure 5. Conversely, in Low AQ score individuals the pattern of connections entering into nodes with higher *authority* was less structured, showing connections directed to frontal (PAC r), right temporal (ST r) and left temporal (FU l) regions.

**FIGURE 8:**
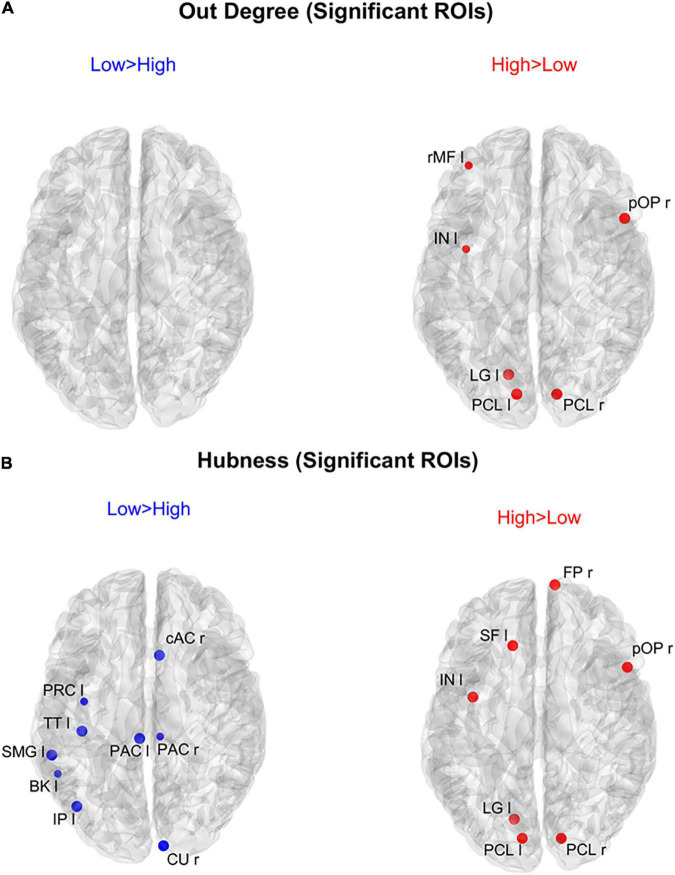
Positions of the ROIs which exhibited a significant difference in the *out degree* index [upper panels **(A)**] or in the *hubness* index [lower panels **(B)**] between the two groups (*p*-value < 0.05, Bonferroni corrected). The left panels in blue (Low > High) display the ROIs having significantly higher centrality index in the Low AQ score Group compared to the High AQ score Group. The right panels in red (High > Low) display the ROIs having significantly higher centrality index in the High AQ score Group compared to the Low AQ score Group. The significant ROIs are shown as simple dots and represent regions from which important information originates. Three levels of dots’ size have been adopted: the larger the dot size, the more significant the centrality difference. For the panels where no dot appears over the brain map (i.e., *out degree* for Low > High), the constraint of significance was not satisfied by any of the 68 ROIs.

**FIGURE 9:**
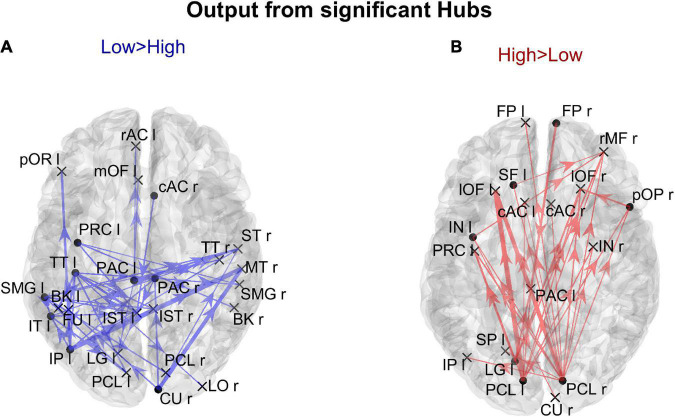
Representation of the connection differences *exiting* from the ROIs which exhibited significant differences of *hubness* between the two groups. The left panel in blue [Low > High **(A)**] displays the connection differences exiting from the “Low > High” *hubness* ROIs (the ROIs shown in the left lower panel in Figure 8), for connections higher in the Low compared to the High AQ score Group. The right panel in red [High > Low **(B)**] displays the connection differences exiting from the “High > Low” *hubness* ROIs (the ROIs shown in the right lower panel in Figure 8), for connections higher in the High compared to the Low AQ score Group. The plotted connections run from the ROIs with significant *hubness* (marked with a dot) toward generic input ROIs (marked with a cross). The thickness of each link varies according to the value of the connection difference. Three levels of thickness are adopted, with a higher thickness indicating a larger connectivity difference.

Figure 8 shows the positions of the cortical ROIs that exhibited a significant difference (Bonferroni corrected) in the *out degree* (upper panels) and *hubness* (bottom panels) indices between the two groups. As shown in the right panels, in the High AQ score Group, both the above-mentioned centrality measures were significantly higher (compared to the other group) in the occipital PCL regions of both hemispheres and in the occipital left LG region. Moreover, some frontal regions also exhibited increased *hubness*, a result apparently in contradiction with previous figures. However, as will be clarified when discussing Figure 9 below, connections originating from these hubs were less significant than those originating from the occipital regions. The Low AQ score Group exhibited an appreciable increase in the *hubness* of parietal and temporal regions, especially in the left hemisphere, whereas no significant increase emerged from the *out degree* index. It is interesting to note that also an occipital region (the CU right) exhibited an increased *hubness* in the Low AQ score Group.

The results illustrated in Figure 8 are further clarified in Figure 9, which shows the connection differences exiting from the nodes with significant higher *hubness* in either group. Once again, a clear bottom-up pattern is evident in the High AQ score Group. It is worth noting that, in this group of individuals, the front-parietal regions with increased *hubness* (i.e., the SF l, FP r, pOP r, and IN l) generated only weak output connections (when compared to the other group). These were sufficient to make the *hubness* of these ROIs significantly higher, without altering the general bottom-up pattern of the overall circuitry. In fact, much stronger connections exited from the two PCL regions, defining a clear bottom-up trend. The pattern of connections originating from significant hubs in the Low AQ score Group were mainly directed from temporal and parietal left regions to the right ones, with some connections also directed downwards to the occipital nodes. As anticipated above, also the right CU exhibited a clear bottom-up function in this group, while, in agreement with Figure 7, the right temporal regions received most of the significant connectivity originating from the hubs. It is worth noting that connections toward frontal regions were less significant in this group.

## Discussion

The present paper analyzes the differences in brain connectivity between two groups of non-clinical individuals who differ in the degree of autistic traits (low vs. high), as classified based on the Autistic Quotient (Baron-Cohen et al., 2001) score. Results have two main important aspects of interest. First, we confirm that autistic traits can be observed within a wide spectrum encompassing both clinical and non-clinical populations. Specifically, the degree of autistic traits clearly differs in the non-clinical population between low and high AQ scores. Second, we show that these differences can be quantified as alterations in brain connectivity. In particular, we show that Granger Causality, computed from neuroelectric signals reconstructed in the cortex (Deshpande and Hu, 2012; Stokes and Purdon, 2017; Cekic et al., 2018), together with indices taken from the Graph Theory (van Wijk et al., 2010; Minati et al., 2013; Farahani et al., 2019), can represent a valuable tool to characterize differences in brain networks and deepen our analysis of the neurobiological bases of brain disorders. Further, we confirm a previous hypothesis (Tarasi et al., 2021, 2022) that individuals with higher autistic traits are characterized by more evident bottom-up mechanisms for processing sensory information.

A critical point may be the selection of the threshold used to discriminate between the two classes. Despite the inherent arbitrariness of the choice, we used as a discriminative threshold the average AQ score obtained in a nonclinical population from the large-sample work of Ruzich et al. (2015), and this seems the most natural choice. Moreover, using this value, the present population of 40 subjects is subdivided in 19 and 21 subjects, i.e., the threshold we chose is quite proximal to the median of the considered population. It is worth noting that similar approaches of partitioning the sample around a threshold have been used previously in the literature (Alink and Charest, 2020).

In the following, we will first analyze methodological issues, then the neurophysiological significance of the obtained results will be explored. Finally, limitations of the present study will be analyzed.

### Granger causality

In this work, we have chosen temporal Granger causality as a tool to reconstruct brain connectivity from EEG data. This measure mathematically represents the impact that knowledge of an upstream signal can have on the prediction of a downstream temporal signal. Thus, it represents a causal directed index of connectivity. Indeed, Granger Causality is widely employed in neuroscience today (Deshpande and Hu, 2012; Seth et al., 2015; Stokes and Purdon, 2017; Cekic et al., 2018). Moreover, in a recent paper, using artificial signals produced by a neurocomputational model as ground truth, we demonstrated that the Granger Causality overcame other functional connectivity estimators in terms of accuracy and reproducibility (Ricci et al., 2021). This method has evident computational advantages compared with other suitable methods [such as Transfer Entropy, see Ursino et al. (2020)].

The analysis was initially performed (see Section “Analysis of the complete connectivity matrix”) on the complete normalized connectivity matrix, to show the main characteristics of the Granger flow in the two groups. Then, to improve the significance of the results, we considered only connections which exhibited a significant statistical difference between the two populations, thus working with a sparse matrix (i.e., all connections which did not show statistically significant differences between the two groups were set at zero). In other terms, the graphs in Section “Analysis on the sparse connectivity matrix” do not represent the overall connectivity patterns, but rather highlight the differences between the two populations.

The connectivity matrices so obtained were then used to compute some indices taken from Graph Theory.

### Graph theory

Several studies using Graph Theory in ASD have appeared in recent years: most of them suggest that ASD individuals exhibit alterations in modularity (i.e., densely connected modules that are more segregated), in global efficiency (i.e., average path length required to go from one node to another), in betweenness (the capacity of a node to connect to other nodes) or in connection density (Rudie et al., 2012; Redcay et al., 2013; You et al., 2013; Keown et al., 2017; Chen et al., 2021). EEG and MEG connectivity studies using graph analysis generally report autism to be associated with sub-optimal network properties (less clustering, larger characteristic path, and architecture less typical of small-world networks) (Barttfeld et al., 2011; Tsiaras et al., 2011; Boersma et al., 2013; Peters et al., 2013; Leung et al., 2014; Takahashi et al., 2017; Soma et al., 2021). This, in turn, results in a less optimal balance between local specialization (segregation) and global integration (Sporns and Zwi, 2004). Although of particular significance, we think that these indices do not consider the fundamental problem of directionality in the processing pathway and the different importance that bottom-up and top-down connectivity plays in several brain processing.

Accordingly, an essential novelty of the present study concerns the use of some specific centrality indices (*in degree*, *out degree*, and above all, *hubness* and *authority*) to characterize group differences in network directionality. The basic idea is that the directionality of the processing streams plays a major role in determining group differences (at least for what concerns autistic traits), rather than other indices like betweenness, path length, or clustering, more frequently adopted in the characterization of brain networks. In particular, by considering macro-regions and sparse connectivity matrices, these indices provided highly significant statistical differences and provided a precise scenario to distinguish the two groups.

### Connectivity among macro-areas (lobes)

The connectivity analysis was performed at two levels. First, we concentrated on the connectivity among macro-regions (lobes) of the cortex, the frontal, parietal, temporal, and occipital zones, to discover the main traits of connectivity differences.

This analysis confirms the result of a previous preliminary study (Tarasi et al., 2021), i.e., individuals with higher autistic traits exhibit stronger outgoing connections from the occipital regions and stronger incoming connections toward frontal areas (i.e., bottom-up) compared with those observed in individuals with lower autistic traits. In addition to confirming the results of our previous study, as a new significant result of the present study we propose that two other centrality measures, i.e., *hubness* and *authority*, allow for a finer discrimination of connectivity directionality. The reason for this improvement will be critically analyzed in the next section. If these two measures are used, significant statistical differences can be observed to characterize the directionality of the connections in High AQ score vs. the Low AQ score individuals. In particular, using sparse matrices statistically significant differences were evident between the *hubness* of the occipital regions in the two classes, with much stronger *hubness* for individuals with high autistic traits. Looking at *authority*, a significant increase in the *authority* of the frontal region was observed in the group with higher autistic traits.

The same patterns were confirmed by computing (from the sparse matrices) the connectivity among the macro-regions and plotting only those which exhibited a significant statistical difference. As shown in Figure 5, increased bottom-up connectivity from occipital to frontal regions was evident in individuals with high autistic traits.

### Connectivity among individual regions of interests

Besides connectivity analysis at lobe level, we performed connectivity analysis at single ROI level. To this aim, *centrality indices* were computed by considering all the 68 ROIs in the Desikan–Killiany atlas. It is interesting that the results obtained on the overall connectivity matrix and on the sparse matrix provide similar indications, emphasizing the presence of bottom-up connections in the high-score group and left-right connections in the low-score group. However, analysis performed on the overall connectivity matrix did not reach a significant level, whereas a greater significance was obtained from sparse matrices. For this reason, in the following we will mainly refer to the results of sparse matrix.

An important result of our study is that hubness and authority provided more significant differences compared with in degree and out degree, respectively; hence we suggest that these indices should be used to characterize the flow in a network of multiple ROIs. In particular, by comparing in degree vs. authority in Figure 6 we can observe that the results are quite similar for what concerns the High AQ score Group (authority produces just one more significant frontal node compared with in degree), whereas significant differences can be observed in the Low AQ score Group (no significant node is evident if in degree is used, compared with five nodes using authority). Consequently, authority allowed the detection of a clear left to right connectivity in the Low AQ score Group. Similarly, only moderate differences can be observed using hubness vs. out degree in the High AQ score Group (Figure 8, hubness detects two additional regions in the frontal cortex, allowing a better analysis of top-down influences). Also in this case, hubness provided a significant improvement compared with the out degree in the Low AQ score Group (nine significant ROIs are detected by hubness, mainly located in left and medial parietal and temporal regions, vs. no significant region by the out degree). These differences suggest that the overall graph is more complex in the Low AQ score Group compared with the High AQ score one, requiring more sophisticate indices for detecting the flow of transmitted information.

To understand why authority and hubness are more powerful compared with in degree and out degree, we remind that authority does not only take into account the number and strength of the connections entering a node but also weights these connections by the *hubness* of the upstream nodes. Similarly, *hubness* does not only take into account the number and strength of connections exiting from a node but also weights these connections by the *authority* of the downstream nodes. Of course, these measures need to be computed together *via* recursive formulas, as illustrated in Eqs 6, 7. Briefly, the importance of the information exiting from a node (or the importance of the information entering into a node) is not simply the sum of its output connections (or the sum of the input connections), but also depends on the role played by the sending nodes (or by the receiving nodes). For instance, a connectivity of value 0.04 reaching an almost completely isolated node (one which does not send information to others nodes in the network) can be scarcely important compared with a connection of value 0.02, which reaches a crucial node. Hubness is able to quantify this difference compared with a simple sum of outgoing connectivity. Similarly, authority is more able to summarize the effective significance of the incoming flow compared with the simple sum of entering connections.

Using these indices, we then mapped the stronger connections that exited from ROIs with higher *hubness* and entered into the ROIs with greater *authority*. These results computed on each ROI extend the lobe analysis to several aspects: (i) The main hubs for High AQ score individuals were located in the left and right PCL regions. A pattern of bottom-up connections emerging from these two regions seems to be the dominant feature that characterizes this group. Left and right PCL are the ROIs in which the primary visual cortex is located. These areas handle the transmission of incoming visual inputs from the thalamus to higher-order processing regions. The enhanced bottom-up signaling arising from this site resembles the pattern observed in individuals with clinical form of autism characterized by hyper-engagement of sensory regions (Jao Keehn et al., 2017, 2019) that could underpin the sensory and visuospatial peculiarities typically observed in ASD (Mottron et al., 2006; Samson et al., 2012). (ii) The leading *authorities* for High AQ score individuals were located in the frontal and prefrontal regions, particularly in the left and right lOF. These two ROIs encapsulate frontal sites involved in high-level mechanisms such as emotional regulation, decision-making and social cognition (Rolls, 2004). Crucially, these domains tend to be altered in ASD individuals. Excessive information inflow in brain areas related to emotional and social processing could be implicated in the difficulty to manage complex and multifaceted social interactions typically observed in this spectrum. This could also explain why ASD individuals tend to prefer less social-demanding environments as they are linked to a lower risk of over-stimulation. (iii) The previous connections were distributed bilaterally, from both PCLs to both homolateral and contralateral frontal hemispheres. (iv) Conversely, the pattern of connectivity in Low AQ score individuals exhibited a broader and less defined distribution, involving several connections in the temporal, parietal, and occipital lobes, with hubs mainly located in the left hemisphere and a direction from left to right. This suggests that the pattern of inter-areas communication in low-AQ individuals is more distributed and varied and not rigidly channeled into narrow pathways.

We remind, however, that these connectivity patterns reflect *differences* between the two groups, hence a relative role in one population vs. the other, not the absolute impact that connections have on the overall brain network. In other words, it is possible that some strong connections did not appear in our graph since they were equally relevant in both populations, hence without significant difference (this is the reason why the overall connectivity matrix provides less significant results). Moreover, we remind that trials were performed at rest. Thus, the examined connectivity reflects differences in a resting state.

In general, the present results support the findings obtained in our previous study on a smaller population (Tarasi et al., 2021), even though the exact position of the ROIs representing the increased bottom-up connectivity is not identical. In our previous study, we observed increased connectivity from the right PCL and the left LG (instead of the left PCL as found here). Still, these differences can be explained based on minor variances in source reconstruction and grouping among proximal voxels. Moreover, in our previous study, the bottom-up connectivity in High-AQ score individuals was especially evident in the right hemisphere (particularly toward the right rMF, a region that still plays e significant role among the authorities in the present study). In contrast, this connectivity seems to be more bilaterally distributed in the current results.

These results support the idea that the brain network in individuals with higher autistic traits vs. individuals with lower autistic traits is not characterized by a general reduction in connectivity (as hypothesized in some theorizations) but rather that mixed patterns of under- and over-connectivity can be appreciated. Over-connectivity is evident in the fronto-posterior axis, involving bottom-up influences, whereas hypoconnectivity involves many tempo-parietal regions, especially in the left hemisphere.

### Neurophysiological meaning

Several hypotheses on brain connectivity in ASD have been formulated in past years, with apparently contradictory outcomes: while some authors hypothesized more robust connectivity in ASD, others reported reduced connectivity (see Section “Introduction”). These contradictions, however, can be reconciled by thinking that differences between control and individuals within the autistic spectrum can especially reflect a directionality in the connections rather than the number and total strength of edges in the overall network. Furthermore, a mixed pattern of increased connectivity among some regions and decreased among others probably characterizes the autistic brain. Directionality in the connectivity patterns, in turn, may reflect a hierarchical organization of the processing stream, with bottom-up connections (especially from the occipital towards the frontal lobes) involved in sensory processing and top-down connections reflecting context modulation, and prior knowledge, planning, and attention. This connectivity organization agrees with the so-called predictive coding theory, which assumes that environmental and internal signals are joined together to form a unified model of reality. In particular, the predictive coding theory of ASD (Van de Cruys et al., 2014; Tarasi et al., 2022) hypothesizes that ASD people do not form accurate predictions of the external environment since sensory information supersedes the internal expectation. Our results support this theory, showing that differences in bottom-up connectivity (hence, in the impact that sensory input can have on the global internal model) are stronger in individuals with higher autistic traits, even within a population of healthy individuals.

### Limitations of the present study

A limitation of the present study may be the limited sample size (19 vs. 21 participants). Actually, this number is in line with (and in many cases higher than) the sample employed in published works that use similar experimental procedures and investigate similar phenomena [see Carter Leno et al. (2018) and Harris et al. (2021)]. However, the complexity of the analysis performed and, in particular, the study accomplished on the complete connectivity matrix, reveal the necessity of a larger number of participants to achieve statistically more solid results. Hence, future studies on a large cohort can allow a more detailed comprehension of the problem.

In this study, we did not include participants with a diagnosis of ASD, hence we cannot be confident that the present results would stand up also in a clinical population. However, the results obtained go exactly in the direction hypothesized by theoretical and empirical work on connectivity features in clinical ASD. Moreover, substantial behavioral (Alink and Charest, 2020), genetic (Bralten et al., 2018), and neural (Massullo et al., 2020) evidence suggests that ASD is a continuum of conditions ranging from trait-like expression to the diagnosed clinical form of autism. Of course, additional studies on a clinical population are required to definitely support the present initial results and definitely validate the hypothesis of a continuous spectrum ranging from normality to ASD.

An interesting point concerns the relationship between the Granger connectivity, evaluated in this study, and the structural connectivity (i.e., the physical traits that connect brain regions, generally estimated by diffusion-weighted imaging). Some studies (e.g., Hermundstad et al., 2013) have shown that there is significant overlap between neuroanatomical connections and correlations of functional brain signals. Conversely, other recent studies of our group, using neural mass models as a ground-truth, showed that in some conditions the two aspects may differ, as a consequence of non-linear phenomena (Ursino et al., 2020, 2021; Ricci et al., 2021). Hence, it is still unclear how the brain network interacts during specific tasks or at rest, accounting for all structural and functional aspects in terms of causality, given the many nonlinear dynamics that characterize brain functioning. Moreover, the present results show some connections crossing the midline. Regarding this point, although the connections traveling through the corpus callosum typically connect homotypic areas, a substantial number of traits connecting heterotypic areas in the two cerebral hemispheres have been observed (e.g., De Benedictis et al., 2016). Of course, without structural data, it remains difficult for the current study to formulate more precise hypotheses about this issue.

Finally, in the present study we have observed differences in bottom-up and top-down connectivity in the two groups. Works in the literature emphasize that these connections can be implicated in sensory processing, especially in multisensory conditions (Choi et al., 2018) or after sensory deprivation (Yusuf et al., 2022). Furthermore, several studies suggest that atypical sensory processing is a common characteristic of ASD and that sensory traits have important implications in the developmental phase of this pathology (Marco et al., 2011; Robertson and Baron-Cohen, 2017). The present experiments were performed in a resting condition, so it would be difficult to make strong inferences about sensory processing from the current data. Further studies, examining the response to sensory stimuli, are required to test whether these neural signatures of autistic traits (more bottom-up processing in high AQ score, more top-down processing in low AQ score) have an impact at the behavioral level, for example to explain the observed differences in sensory profile.

## Data availability statement

The raw data supporting the conclusions of this article will be made available by the authors, without undue reservation.

## Ethics statement

This study was conducted according to the guidelines of the Declaration of Helsinki and approved by the Bioethics Committee of the University of Bologna (protocol code 201723, approved on August 26, 2021). Informed consent was obtained from all participants involved in the study. The patients/participants provided their written informed consent to participate in this study.

## Author contributions

MU, VR, and LT contributed to the conception and design of the study. LT and VR collected the data. MS, GR, and EM developed the software for the analysis, collected the results, and prepared figures. MU wrote the first draft of the manuscript. MS wrote sections of the manuscript. EM reviewed all the manuscript. All authors contributed to the results interpretation, manuscript editing, and read and approved the submitted version.
